# Assessment, reliability, and validity of trichoscopy in the evaluation of alopecia in women

**DOI:** 10.1016/j.ijwd.2021.02.002

**Published:** 2021-02-16

**Authors:** Najam U Saqib, Yasmeen Jabeen Bhat, Iffat Hassan Shah, Inaam Haq, Reeta Devi, Aaqib Aslam Shah, Faizan Younis Shah

**Affiliations:** a Department of Dermatology, Venereology and Leprosy, Government Medical College, Srinagar, University of Kashmir, Jammu and Kashmir, India; b Department of Social & Preventive Medicine, Government Medical College, Srinagar, University of Kashmir, Jammu and Kashmir, India

**Keywords:** Trichoscopy, Alopecia, Female pattern hair loss, Telogen effluvium, Scarring alopecia, Sensitivity of trichoscopy

## Abstract

**Background:**

Alopecia in women is generally difficult to diagnose clinically. Trichoscopy may help make the correct diagnosis in doubtful cases.

**Objective:**

The aims of the study were to assess the trichoscopic features of different types of alopecia in women, the reliability of trichoscopy vis-à-vis clinical findings, and the validity of trichoscopy in cases with a doubtful clinical diagnosis.

**Methods:**

A hospital-based observational, cross-sectional study was carried out on female patients with alopecia. A trichoscopic diagnosis was made and correlated with a clinical diagnosis. The validity of trichoscopy in doubtful cases was evaluated by reporting the sensitivity, specificity, positive predictive value, negative predictive value, and diagnostic value.

**Results:**

On trichoscopy, increased hair diameter diversity > 20%, single-hair follicular unit, vellus hair, peripilar sign, and focal atrichia were commonly seen in female pattern hair loss. In telogen effluvium, there was a scarceness of specific findings. In cicatricial alopecias, loss of follicular ostia, erythema, white macules, blue-gray dots, white dots, tufted hair, and keratotic follicular plugging were observed. A good agreement between trichoscopy and clinical diagnosis was found (Cohen’s Kappa = 0.824; 95% confidence interval, 0.756–0.892). The validity of trichoscopy in doubtful cases was evaluated using the validity parameters, which were high in all alopecias.

**Limitations:**

Histopathology testing was not done in all patients.

**Conclusion:**

Trichoscopy helped reach a definitive diagnosis in patients in whom the clinical diagnosis was doubtful. Thus, trichoscopy is a sensitive and specific investigation that can be valuable in women with alopecia.

## Introduction

Baldness, especially in women, is not acceptable to most people in today’s society. In contrast with existing attitudes toward baldness in men, society generally regards hair loss as abnormal for women (Messenger et al., 2016a, Messenger et al., 2016b). By standard definition and generalization, alopecia is observed with 50% loss of the native or original hair density (Messenger et al 2010). Alopecia can be categorized as cicatricial (scarring) or noncicatricial (nonscarring). The cause of alopecia in women is relatively more difficult to assess clinically, and clinical diagnosis is mostly challenging. Thus, a better understanding of alopecia via a trichoscopic evaluation can be a welcome advancement. Trichoscopy is a new method to diagnose hair loss using dermoscopy of the hair, scalp, eyebrows, and eyelashes to visualize and measure hair at high magnification (Olszewska et al., 2008). Lacarrubba et al. (2004) first described videodermoscopic features of alopecia areata (AA). In 2006, the term “trichoscopy” for hair and scalp videodermoscopy in hair loss diagnostics was first used (Ross et al., 2006). Trichoscopic evaluation of the scalp is based on the study of follicular, interfollicular, and perifollicular hair shaft patterns and hair signs.

This study aimed to assess the trichoscopic features of different types of alopecia in women, the reliability of trichoscopy vis-à-vis clinical findings, and the validity of trichoscopy in cases with a doubtful clinical diagnosis.

## Methods

A hospital-based observational, cross-sectional study was carried out on female patients who visited the outpatient department of dermatology on a regular basis with alopecia. For this study, alopecia was defined as visible thinning or loss of hair from the scalp. Institutional ethical clearance was obtained prior to the study, and written informed consent was received from each participant or her parent/guardian in the case of minors. We calculated the minimum sample size by assuming the prevalence proportion of hair loss type to be 15% with 5% absolute error at a 95% confidence level based on previous studies. Given these parameters, the minimum number of positive cases needed was 196. This was calculated using nMaster, which uses the formula n = (3.84 × p × (1 − p))/d^2^ where p is the proportion and d is the absolute error. The required sample size for the study was taken as 200.

Twenty patients with alopecia were chosen per day. For each day, a random number (n) was generated by the RANDBETWEEN function in Microsoft Excel. The nth female patient received an explanation of the aims and objectives of the study and, after providing proper consent, was included in the study. The study included female patients of all age groups who gave consent for participation in the study. Uncooperative children, as well as pregnant and lactating women were excluded from the study.

Patients’ clinical history was taken, and complete physical, systemic, and mucocutaneous examination was done. The history and clinical findings were recorded on a specially designed proforma, and a clinical diagnosis was made. Routine hematological and biochemical laboratory tests, thyroid function tests, antinuclear antibody titer, gonadal hormonal profile, potassium hydroxide mount, and ultrasounds of the abdomen and pelvis were performed when indicated. Standard textbook criteria were followed while making a diagnosis (Messenger et al., 2016a, Messenger et al., 2016b). After the diagnosis, trichoscopy was performed on the patient using a USB-connected video dermoscope (AM7515MZT Dino-Lite Edge, 220×) in both nonpolarized and polarized modes at magnifications ranging from 20× to 220×. Seventy-percent alcohol was used as the contact medium.

In cases of diffuse and patterned hair loss, to maintain the uniformity of the procedure, a trichoscopy was done at five fixed sites: at the mid parting, 3 cm from the anterior hair line of the frontal scalp, temporal scalp bilaterally (2 cm lateral to the mid-pupillary line), vertex, and occipital (5 cm below the vertex) regions of the scalp. This also gives a global assessment of the scalp (Jayasree et al., 2021). The different trichoscopic features were seen at these sites, compared with each other, analyzed, and recorded.

In cases of patchy hair loss, trichoscopy was done at the periphery and center of the alopecia patch, and the trichoscopy findings were recorded. Photographs of the different trichoscopic features were taken with the video dermoscope when required. Trichoscopic trichogram of pulled hair to identify the hair roots was done wherever required.

A trichoscopic diagnosis was made and correlated with the clinical diagnosis. In case of discordance between the two diagnosis, the patient was labeled as a doubtful case, and biopsy was performed to confirm the diagnosis.

### Statistical analysis

The data were entered in a Microsoft Excel spreadsheet, and the categorical variables were summarized as frequencies and percentages. Agreement between trichoscopy and clinical diagnosis was evaluated using Cohen’s Kappa. The validity of trichoscopy in doubtful cases was evaluated by reporting the sensitivity, specificity, positive predictive value (PPV), negative predictive value (NPV), and diagnostic accuracy. EpiInfo7.2 was used to evaluate the validity parameters. Sensitivity, specificity, PPV, NPV, and diagnostic accuracy were reported as percentages along with their 95% confidence interval (CI). Cohen’s Kappa was calculated using MedCalc 17.6. Two-sided *p*-values were reported, and *p* < .05 was considered statistically significant.

## Results

The study included a total of 200 female patients. The age at presentation ranged from 4 to 70 years, and the median age was 23 years (interquartile range, 19–30 years). Most belonged to the age group of 20 to 29 years. The age at the time of hair loss onset in the study patients ranged from 1 to 59 years, with a median age of onset of 21 years (interquartile range, 17–28 years).

Clinically, 57% of patients were suspected as having female pattern hair loss (FPHL), 16% as AA, 13.5% as telogen effluvium (TE), 3.5% as trichotillomania (TTM), 3% as pseudopelade of Brocq (PoB), 3% as traction alopecia (TA), 1.5% as folliculitis decalvans (FD), 1% as lichen planopilaris (LPP), 1% as discoid lupus erythematosus (DLE), and 0.5% as tinea capitis. A pull test was positive only in cases with a clinical diagnosis of TE, AA, and FPHL, comprising 21.5% of the total number of cases (n = 43 of 200). In addition, 48.8% of cases of TE, 34.9% of AA, and 7% of FPHL had a positive pull test. Trichoscopic trichogram of the pulled hair showed only telogen hair in all cases of TE and a majority of cases of FPHL. Telogen and dystrophic hair were seen in cases of AA.

On trichoscopy, the most common diagnosis was FPHL in 57.5% of cases (n = 115 of 200), followed by AA in 17% (n = 34), TE in 13% (n = 26) , PoB in 2.5% (n = 5), TA in 2.5% (n = 5), TTM in 2.5% (n = 5), FD in 1.5% (n = 3), LPP in 1.5% (n = 3), DLE in 1.5% (n = 3), and tinea capitis in 0.5% (n = 1). In doubtful cases with a disagreement between clinical diagnosis and trichoscopic diagnosis, histopathology was carried out, which showed features of FPHL in 7 of 26 doubtful cases, PoB in 6, TE in 5, LPP in 2, TA in 2, FD in 2, and DLE in 1 case, and was inconclusive in 1 case.

**Fig. 1:**
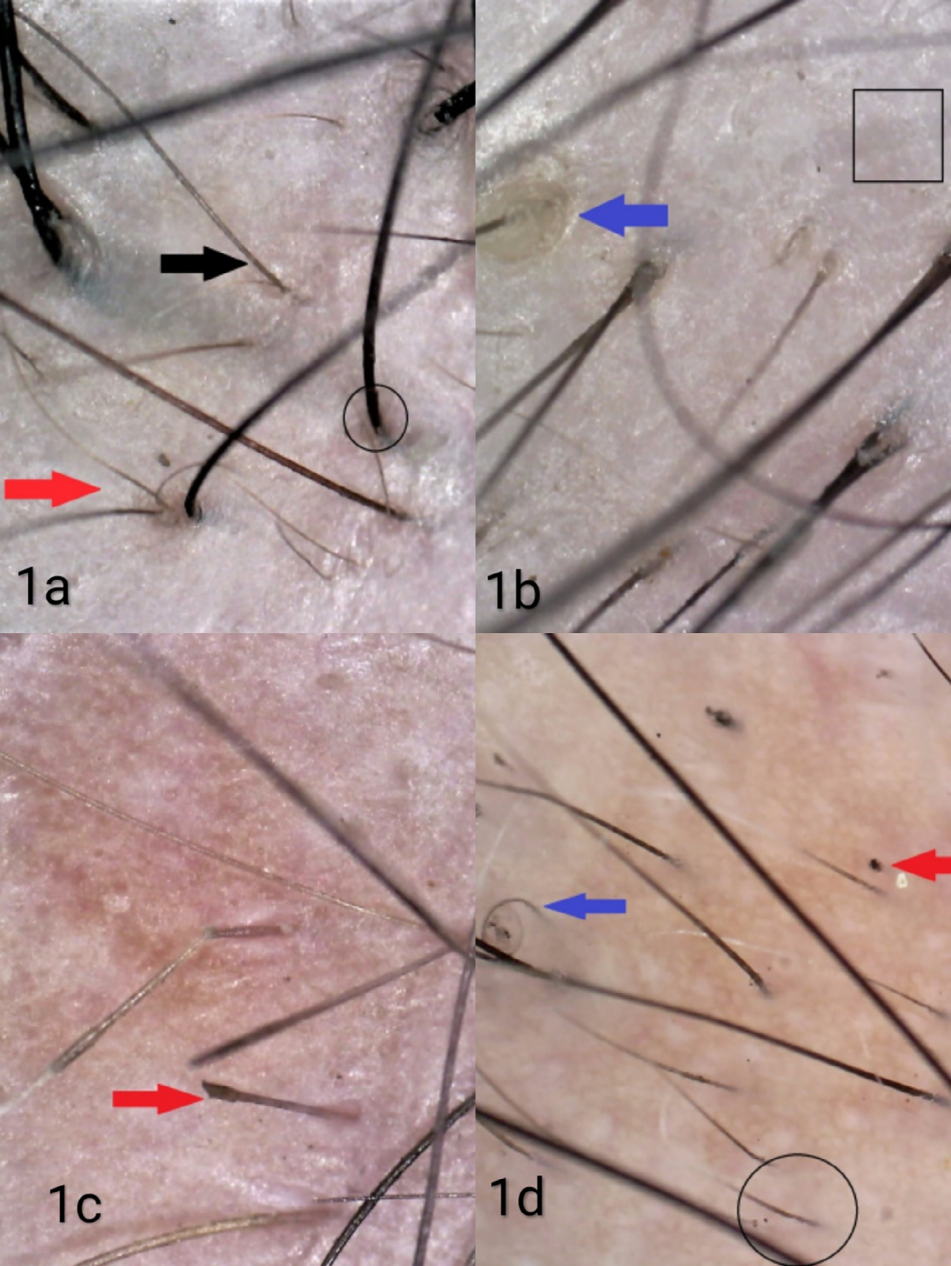
(A) Female pattern hair loss showing hair diameter diversity >20%, vellus hair (red arrow), thin hair (black arrow), and single-hair follicular unit (black circle; 75×, nonpolarized). (B) Female pattern hair loss showing peripilar halo (blue arrow) and focal atrichia (black square; 80×, nonpolarized). (C) Alopecia areata showing exclamation mark hair (red arrow; 80×, nonpolarized). (D) Alopecia areata showing black dot (red arrow), pigtail hair (blue arrow), and upright regrown hair (black circle; 65×, polarized).

**Fig. 2:**
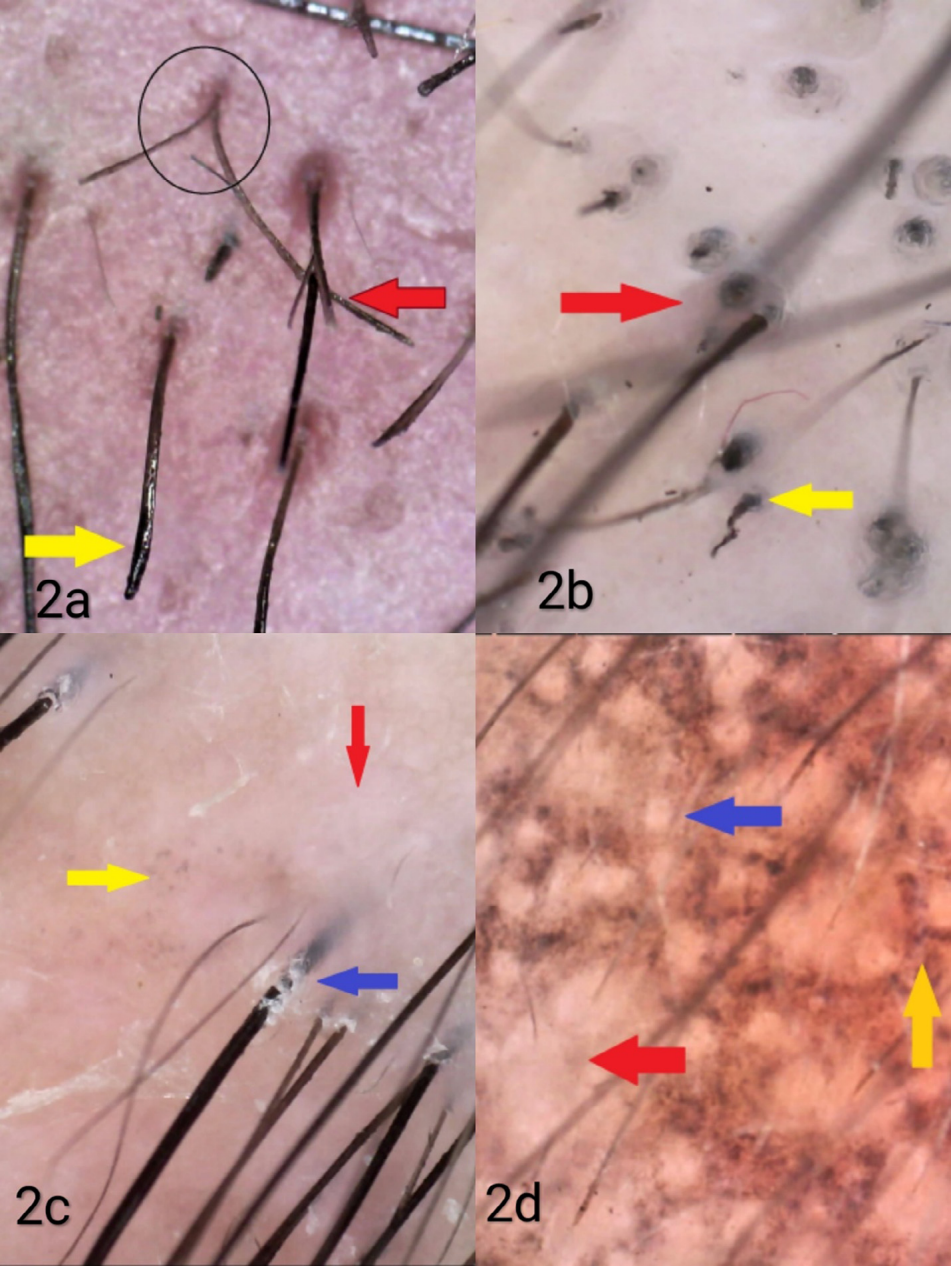
(A) Trichotillomania showing trichotilopsis (red arrow), broken hair of unequal length (yellow arrows), and V sign (black circle; 65×, nonpolarized). (B) Trichotillomania showing flame hair (yellow arrow) and hair dust (red arrow; 70×, polarized). (C) Lichen planopilaris showing white macules (red arrows), peripilar casts (blue arrow), and blue-gray dots (yellow arrow; 70×, polarized). (D) Lichen planopilaris showing perifollicular blue-gray dots (yellow arrow), perifollicular white macules (red arrow), and white dots (blue arrow; 80×, polarized).

All patients with FPHL showed hair diameter diversity > 20%, with a single hair coming out of each follicular opening and thin hair. Other findings were vellus hair (98.3%), peripilar halo (88.7%), yellow dots (28.7%), and focal atrichia (16.5%). The trichoscopic features of other nonscarring alopecias are shown in Table 1 (Figs. 1, 2A, and 2B).

**Table 1:** Trichoscopic features in different types of nonscarring alopecias.

Trichoscopic features		n	%
Female pattern hair loss (n = 115)	Hair diameter diversity > 20%	115	100
	Thin hair	115	100
	Single hair coming out of each follicular unit	115	100
	Vellus hair	113	98.3
	Peripilar halo	102	88.7
	Yellow dots	33	28.7
	Focal atrichia	19	16.5

Alopecia areata (n = 34)	Yellow dots	25	73.5
	Black dots	22	64.7
	Coudability hair	21	61.8
	Exclamation mark hair	19	55.9
	Broken hair	18	52.9
	Vellus hair	14	41.2
	Pigtail hair	13	38.2
	Regrown hair	12	35.3
	Tulip hair	2	5.9

Telogen effluvium (n = 26)	Upright regrowing hair	25	96.2
	Single hair coming out of each follicular unit	3	11.5

Traction alopecia (n = 5)	Peripilar cast	4	80
	Single hair coming out of each follicular unit	4	80
	Vellus hair	1	20

Trichotillomania (n = 5)	Broken hair of different lengths	3	60
	Tulip hair	3	60
	V sign	3	60
	Flame hair	3	60
	Split ends	3	60
	Black dots	2	40
	Peripilar hemorrhages	2	40
	Hair dust	2	40
	Pigtail hair	2	40
	Single hair coming out of each follicle	2	40
	Perifollicular scaling	1	100

Tinea capitis (n = 1)	Comma hair	1	100

**Fig. 3:**
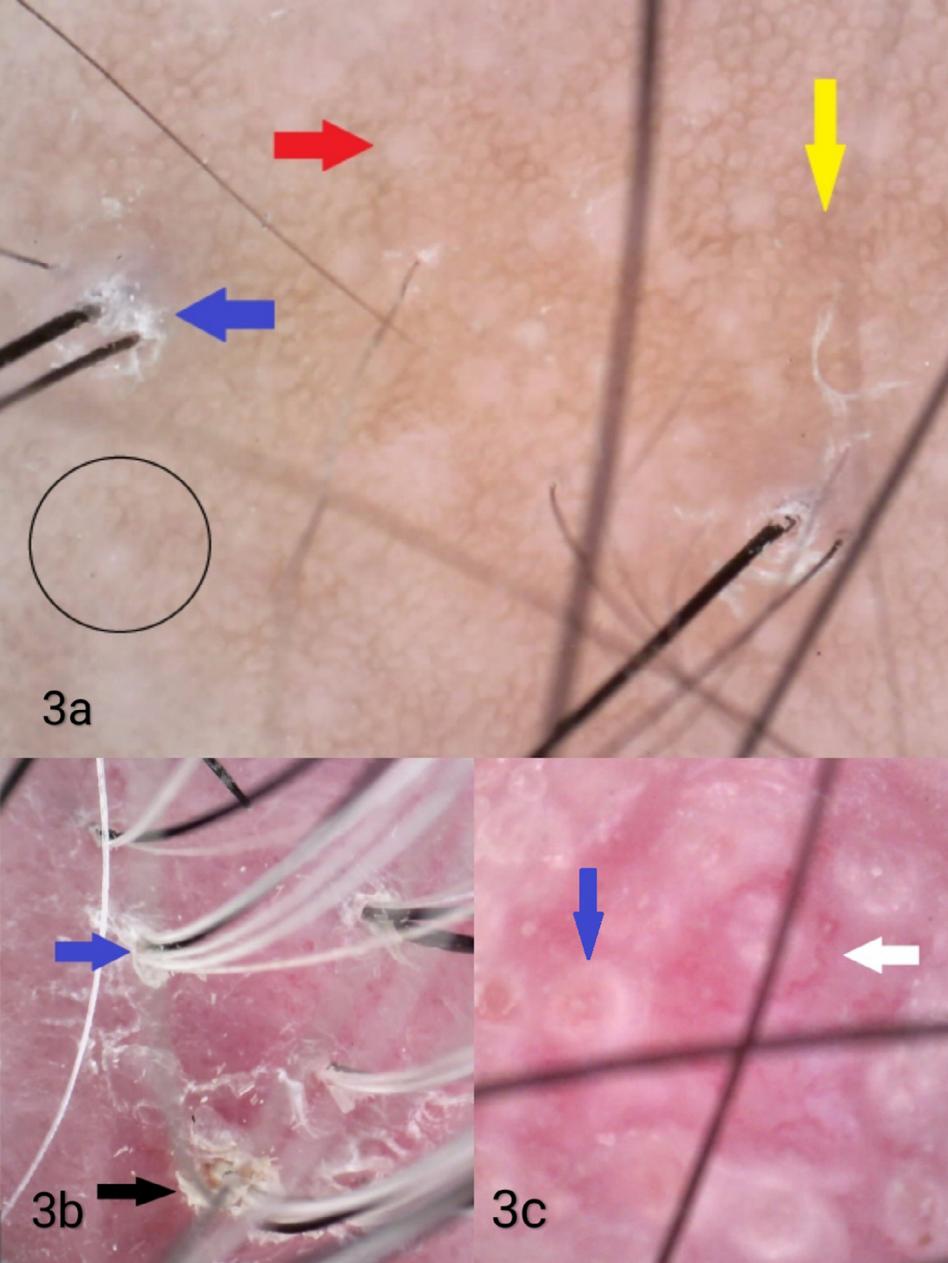
(A) Pseudopelade of Brocq showing white dots (red arrow), honeycomb pigmentation (yellow arrow), perifollicular scaling (blue arrow), and absence of follicular ostia (black circle; 72×, polarized). (B) Folliculitis decalvans showing polytrichia/tufted hair (blue arrow) and perifollicular scaling (black arrow; 70×, polarized). (C) Discoid lupus erythematosus showing follicular plugging (blue arrow) and branching vessels (white arrow; 147×, polarized).

All patients with LPP showed perifollicular erythema (PE), perifollicular scaling (PS), perifollicular white macules, and blue-gray dots. The trichoscopic findings in other scarring alopecias are shown in Table 2 (Figs. 2C, 2D, and 3).

**Table 2:** Trichoscopic features in different types of scarring alopecias.

Trichoscopic features		n	%
Lichen planopilaris	Perifollicular erythema	3	100
	Perifollicular scaling	3	100
	Perifollicular white macules	3	100
	Blue grey dots	3	100
	Absent follicular openings	2	66.7
	White dots	1	33.3
	Peripilar cast	1	33.3
	Honeycomb pigmentation	1	33.3

Pseudopelade of Brocq	Absent follicular openings	5	100
	Perifollicular scaling	5	100
	Perifollicular white macules	5	100
	White dots	3	60
	Yellow dots	2	40
	Perifollicular erythema	2	40
	Honeycomb pigmentation	1	20
	Vellus hair	1	20

Folliculitis decalvans	Polytrichia	3	100
	Perifollicular erythema	3	100
	Perifollicular scaling	3	100
	Perifollicular white macules	3	100
	White dots	2	66.7
	Absent follicular openings	2	66.7

Discoid lupus	Keratotic plugs	3	100
	Absent follicular openings	3	100
	Perifollicular erythema	3	100
	Perifollicular scaling	3	100
	Perifollicular white macules	2	66.7
	White dots	2	66.7
	Branching vessels	2	66.7

### Assessing reliability of trichoscopy vis-à-vis clinical findings

To assess the reliability of trichoscopy compared with the clinical findings, Cohen's kappa coefficient was used. In our study, the coefficient for the trichoscopic and the clinical diagnosis was 0.824 (95% CI, 0.756–0.892) and achieved the satisfactory level (Table 3). The diagnosis in doubtful cases based on trichoscopic and histopathological features was compared. There was a good agreement between the histological and trichoscopic diagnosis (Kappa coefficient = 0.858; 95% CI, 0.711–1.00).

**Table 3:** Agreement between trichoscopy and clinical diagnosis.

Trichoscopic diagnosis	Clinical diagnosis										Total
	Female pattern hair loss	Alopecia areata	Telogen effluvium	Lichen planopilaris	Pseudopelade of Brocq	Traction alopecia	Trichotillomania	Tinea capitis	Folliculitis decalvans	Discoid lupus	
Female pattern hair loss	107	2	4	0	0	2	0	0	0	0	115
Alopecia areata	1	30	2	0	0	0	1	0	0	0	34
Telogen effluvium	5	0	21	0	0	0	0	0	0	0	26
Lichen planopilaris	0	0	0	2	1	0	0	0	0	0	3
Pseudopelade of Brocq	0	0	0	0	5	0	0	0	0	0	5
Traction alopecia	1	0	0	0	0	4	0	0	0	0	5
Trichotillomania	0	0	0	0	0	0	5	0	0	0	5
Tinea capitis	0	0	0	0	0	0	0	1	0	0	1
Folliculitis decalvans	0	0	0	0	0	0	0	0	2	1	3
Discoid lupus	0	0	0	0	0	0	1	0	1	1	3
Total	114	32	27	2	6	6	7	1	3	2	200

Cohen’s Kappa = 0.824; 95% confidence interval, 0.756–0.892.

### Assessing the validity of trichoscopy

We evaluated the validity of trichoscopy in doubtful cases using the validity parameters (i.e., sensitivity, specificity, PPV, NPV, and diagnostic accuracy), which were high for most nonscarring and scarring alopecias. The results are shown in Table 4.

**Table 4:** Validity of trichoscopy in cases with doubtful clinical diagnosis taking histopathology as gold standard.

	Sensitivity (95% CI)	Specificity (95% CI)	Positive predictive value (95%CI)	Negative predictive value (95% CI)	Accuracy (95% CI)
Female pattern hair loss	100	94.74	87.5	100	96.15
	(64.57–100)	(75.36–99.06)	(52.91–97.76)	(82.41–100)	(81.11–99.32)

Telogen effluvium	100	100	100	100	100
	(56.55–100)	(84.54–100)	(56.55–100)	(84.54–100)	(87.13–100)

Lichen planopilaris	100	95.83	66.67	100	96.15
	(34.24–100)	(79.76–99.26)	(20.77–93.85)	(85.69–100)	(81.11–99.32)

Pseudopelade of Brocq	83.33	100	100	95.24	96.15
	(43.65–96.99)	(83.89–100)	(56.55–100)	(77.33–99.15)	(81.11–99.32)

Traction alopecia	50	100	100	96	96.15
	(94.53–90.55)	(86.22–100)	(20.65–100)	(80.46–99.29)	(81.11–99.32)

Folliculitis decalvans	100	95.83	66.67	100	96.15
	(34.24–100)	(79.76–99.26)	(20.77–93.85)	(85.69–100)	(81.11–99.32)

Discoid lupus erythematosus	100	100	100	100	100
	(20.65–100)	(86.68–100)	(20.65–100)	(86.68–100)	(87.13–100)

CI, confidence interval.

## Discussion

Clinically, FPHL was the most common diagnosis in our study, followed by AA, TE, TTM, TA, and tinea capitis. Among scarring alopecias, PoB was the most common diagnosis present, followed by FD, LPP, and DLE.

Hair shaft diameter diversity, or anisotrichosis, is the most common feature observed in FPHL and reflects hair miniaturization due to disease (Sewell et al., 2007). Hair shaft diameter diversity >20% was present in the frontal scalp of all cases of FPHL in our study (Bhat et al., 2020, Varma et al., 2020). Thin hair and single-hair follicular units were also seen. Hypopigmented and nonmedullated vellus hair, which is a sign of severe miniaturization, was seen in 98.3% of cases with FPHL (Rakowska et al., 2009).

In our study, 88.75% of cases with FPHL showed the peripilar sign, a brown depressed halo of approximately 1 mm diameter at the follicular opening that correlates with perifollicular inflammation, although it may be difficult to identify in dark skin (Chiramel et al., 2016, Deloche et al., 2004, Zhang et al., 2012). Yellow dots, corresponding to follicular ostia filled with sebaceous material, were found in 28.7% of patients in our study (Ross et al., 2006, Rudnicka et al., 2011). Focal atrichia was found in 16.5% of cases. Rakowska et al. (2009) formulated major and minor trichoscopic criteria for a diagnosis of FPHL. We did not apply these criteria to our patients; however, our findings were comparable with most of the criteria set by the authors (Rakowska et al., 2009).

Out of the 34 cases with AA, yellow dots, representing distended follicular orifices filled with keratin, were found in 73.5% of cases (Gordon et al., 2013, Guttikonda et al., 2016). These correlate with disease severity (Bains and Kaur, 2020, Karadag Köse and Güleç, 2012, Mane et al., 2011). Black dots, formed when pigmented hair is broken at the scalp level, were seen in 64.7% of cases. Exclamation-mark hairs, representing broken hairs with frayed thicker distal ends and thinner proximal shafts, were seen in 55.9% of cases (Inui et al., 2010). Our other findings included coudability hair (i.e., long hair with proximal hair-shaft tapering that may be formed due to a less severe injury to the hair follicle, which continues into an anagen phase and broken hair; Shuster, 1984). Tulip hair (i.e., broken hairs that are not pigmented proximally and have distal ends that look like a tulip) was also seen (Rudnicka et al., 2012). Short, upright vellus hair and thin, twisted vellus hair (known as pigtail hair), the two types of hair suggestive of regrowth in patients with AA, were also seen in these patients (Lacarrubba et al., 2004).

In case of TE, although upright regrowing hairs representing regrowing hair in the remitting phase of TE were the most prominent feature, seen in 96.5% of cases, followed by single follicular unit in 11.5% of cases, there was a scarceness of specific findings (Kowalska-Oledzka et al., 2012). In TA, peripilar casts, single follicular unit, and vellus hair (representing the gradual shortening of hairs follicular miniaturization) were observed (Ocampo-Garza and Tosti, 2018, Tanus et al., 2015). The presence of a peripilar hair cast indicates active traction to the hair (Mathur et al., 2019).

In TTM, the most common findings were broken hair of unequal length, tulip hair, flame hair, V sign, and split ends, followed by black dots, peripilar hemorrhages, hair dust, pigtail hair, and single follicular unit. Most of these signs are evidence of hair pulling (Jain et al., 2013). Hair powder (pigment particles sprinkled near the follicle openings) occurs due to the complete destruction of the hair shaft from mechanical trauma (Ankad et al., 2014, Rakowska et al., 2014). There was only one case of tinea capitis where comma hair and PS was seen on trichoscopy. Comma hairs are broken single hair shafts that curl into a comma-like structure due to the bending of a hair shaft secondary to hair ectothrix parasitation (Slowinska et al., 2008, Waśkiel-Burnat et al., 2020).

In cicatricial alopecias, the absence of follicles was observed in most cases, PS and perifollicular white macules were appreciated in all cases, and PE was seen in all cases except one case of PoB. Findings specific to LPP were perifollicular and interfollicular blue-gray dots seen in all cases, peripilar cast, white dots, and honeycomb pigmentation (Ankad et al., 2013, Elmas, 2019, Sani et al., 2016, Zhang et al., 2012). Findings specific to PoB were yellow dots and honeycomb pigmentation, and polytrichia (tufted hair) was specific to folliculitis decalvans. Findings specific to DLE were keratotic follicular plugging, branching vessels, and white dots (Al-Refu, 2018, Beheshtiroy et al., 2015, Herskovitz and Miteva, 2016, Mathur and Acharya, 2020, Waśkiel-Burnat et al., 2019).

To assess the reliability of trichoscopy vis-à-vis clinical findings, Cohen's kappa coefficient was used and calculated as 0.824 (95% CI, 0.756–0.892), achieving the satisfactory level (Kowalska-Oledzka et al., 2012).

There was 85% concordance between trichoscopic and histopathological diagnosis in our study (Kappa coefficient: 0.858; 95% CI, 0.711–1.00). The sensitivity and specificity of trichoscopy for FPHL was calculated and was high, along with high diagnostic accuracy (Galliker and Trüeb, 2012, Kowalska-Oledzka et al., 2012). The sensitivity and specificity of trichoscopy for TE was 100%, along with a high PPV and NPV. This was in slight contrast to a study by Kowalska-Oledzka et al. (2012), who showed a lower sensitivity of 85%. For all scarring alopecias, the sensitivity, specificity, and diagnostic accuracy were high (Abedini et al., 2016).

## Limitations

We had a limited number of patients in each group of alopecia; thus, we could not assess all trichoscopic features that could have been seen. This was especially true for scarring alopecias. In addition, biopsies were not performed in every patient because the clinical diagnosis harmonized with the trichoscopic diagnosis in most patients; thus, the validity parameters could not be calculated in all types of alopecia, with scalp biopsy the gold standard for comparison.

## Conclusion

In our study, trichoscopy helped with the definitive diagnosis of alopecia in patients for whom the clinical diagnosis was doubtful, which was confirmed by histopathology. Thus, trichoscopy is a noninvasive, sensitive, and specific investigation that is valuable in women with alopecia, the cause of which is otherwise very difficult to assess clinically, and it has a definite role in the diagnosis of cases with an atypical clinical picture. Trichoscopy provides quick detection of scalp and hair disorders with advanced diagnostic accuracy, predicts the course of the disease, and decreases the need for unnecessary biopsies.

## Conflicts of interest

None.

## Funding

None.

## Study approval

The author(s) confirm that any aspect of the work covered in this manuscript that has involved human patients has been conducted with the ethical approval of all relevant bodies.
